# Rapid counting and spectral sorting of live coral larvae using large-particle flow cytometry

**DOI:** 10.1038/s41598-020-69491-0

**Published:** 2020-07-31

**Authors:** Carly J. Randall, Justin E. Speaks, Claire Lager, Mary Hagedorn, Lyndon Llewellyn, Rock Pulak, Julia Thompson, Line K. Bay, David Mead, Andrew J. Heyward, Andrew P. Negri

**Affiliations:** 1 Australian Institute of Marine Science, Townsville, QLD Australia; 2 Hawaiʻi Institute of Marine Biology, Kaneohe, HI USA; 3 Center for Species Survival, Smithsonian Conservation Biology Institute, Front Royal, VA USA; 4 Union Biometrica, Inc., Holliston, MA USA; 5 Australian Institute of Marine Science, Indian Ocean Marine Research Centre, University of Western Australia, Crawley, WA Australia

**Keywords:** Animal physiology, Biological techniques, Developmental biology, Zoology, Ocean sciences

## Abstract

Research with coral embryos and larvae often requires laborious manual counting and sorting of individual specimens, usually via microscopy. Because many coral species spawn only once per year during a narrow temporal window, sample processing is a time-limiting step for research on the early life-history stages of corals. Flow cytometry, an automated technique for measuring and sorting particles, cells, and cell-clusters, is a potential solution to this bottleneck. Yet most flow cytometers do not accommodate live organisms of the size of most coral embryos (> 250 µm), and sample processing is often destructive. Here we tested the ability of a large-particle flow cytometer with a gentle pneumatic sorting mechanism to process and spectrally sort live and preserved *Montipora capitata* coral embryos and larvae. Average survival rates of mechanically-sorted larvae were over 90% and were comparable to those achieved by careful hand-sorting. Preserved eggs and embryos remained intact throughout the sorting process and were successfully sorted based on real-time size and fluorescence detection. In-line bright-field microscopy images were captured for each sample object as it passed through the flow-cell, enabling the identification of early-stage embryos (2-cell to morula stage). Samples were counted and sorted at an average rate of 4 s larva^−1^ and as high as 0.2 s larva^−1^ for high-density samples. Results presented here suggest that large-particle flow cytometry has the potential to significantly increase efficiency and accuracy of data collection and sample processing during time-limited coral spawning events, facilitating larger-scale and higher-replication studies with an expanded number of species.

## Introduction

Coral populations have suffered widespread losses over the last half century as a result of local, regional, and global pressures driven by human activities^1–4^. Research on the early life-history stages of corals is foundational to our understanding of coral bioecology and is an increasingly active area of research as we enter a new era of restoration and adaptation science^5^. Yet, research on the early stages of broadcast spawning corals is severely restricted by spawning events that only occur on a few nights of the year for a given species and location^6,7^. These arduous periods often are defined by long working hours, demands on equipment and resources shared across research groups, and hence missed research opportunities. Much research relies on the painstaking process of counting and hand-sorting individual sub-millimetre diameter oocytes, embryos and larvae into well plates, test tubes, or petri dishes^8^. Furthermore, rapid rates of cleavage^9^ leave little time to work with early developmental stages. Investigations that employ CRISPR/Cas9, for example, require fertilized but not yet cleaving eggs^10^, offering as little as a few hours per year for research. A rapid and reliable method for sorting coral eggs, embryos and larvae, capturing population-level data, and sorting the samples based on condition would enable the scientific community to better capitalise on the narrow window of opportunity for research around coral spawning.

Flow cytometry and fluorescence activated cell sorting are techniques that have been used since the 1960s for the automated and rapid counting, characterisation, and spectral sorting of particles and cells^11–13^. Flow cytometers operate by passing particles in a rapidly flowing stream of water past a focused beam of light and then characterising those particles based on the transmission and scattering of the light beam as measured by detectors. Fluorescent lasers and detectors allow the quantification of fluorescence signals and can measure the spatial pattern of fluorescence throughout the particle^13,14^. Thousands of cells can be measured per second using this technique^15^, and over the last six decades the applications of fluorescence activated cell sorting and flow cytometry have been exceedingly far reaching, from applications in molecular biology and immunology to marine ecology and botany^13,15–18^. Historically, flow cytometry has been limited to small cells and cell clusters; recent technological advancements, however, have enabled the application of flow cytometry to particles more than a millimetre in size^17,19^. New instruments are also equipped with more advanced optical features^13,20^ and have been modified to process live multicellular samples, such as *Caenorhabditis elegans*^19^ and mixed zooplankton^17^, using gentle sorting techniques.

Coral eggs, embryos, and larvae are soft bodied and large in the context of flow cytometry, with diameter frequency peaks of 400–550 µm in multi-species surface slicks^5^. Coral embryos are also naked (i.e., do not have a protective membrane), fragile, and are likely to fragment under the conditions of standard flow cytometry^21^. However, in light of the recent advances that enable gentle sorting of live particles up to ~ 1,500 µm, coral larvae offer an ideal test subject for large-particle flow cytometry. Furthermore, many coral species exhibit intrinsic fluorescence (i.e., autofluorescence) from green fluorescent-like proteins^22^ and some species vertically transmit fluorescent endosymbiotic dinoflagellates through their gametes (e.g. *Montipora* sp.^23^). This intrinsic fluorescence makes them ideally suited to test real-time spectral sorting. Thus, the objectives of the study were to (1) test the viability of live coral embryos and larvae processed with the flow cytometer, (2) evaluate the accuracy of spectral sorting, and (3) test throughput rates of two large-particle flow cytometers that are commercially available.

## Results

### Throughput rates

Throughput rates averaged 0.5 ± 0.2 (SE) particles s^−1^, but were as fast as 3.0 particles s^−1^ and as slow as 0.1 particles s^−1^, and were similar between instruments (Table S1). Throughput rates depended largely on the density of the sample; the highest rate achieved was with a sample density of approximately 200 preserved eggs ml^−1^. By contrast, the slowest rate was measured with a sample density of approximately 1 live larva ml^−1^. The sample deposition container also impacted throughput rates. Deposition into a 48-well plate took more time than into a 6-well plate or petri dish, due to the time required for the movement of the stage into position. The final factor that impacted throughput rates was the sample delivery container. The 15 ml rotating sample cartridge on the COPAS VISION allowed for a gentle delivery of the samples into the flow cell, but due to a constant replenishment of sheath solution, resulted in a decline in sample density over time and a consequent decline in throughput rate. Because the coral samples tested were all approximately the same size, the effect of particle size on throughput rates was not investigated, although in general, smaller particles travel faster through the flow cell than larger particles.

### Larval viability assays

**Figure 1:**
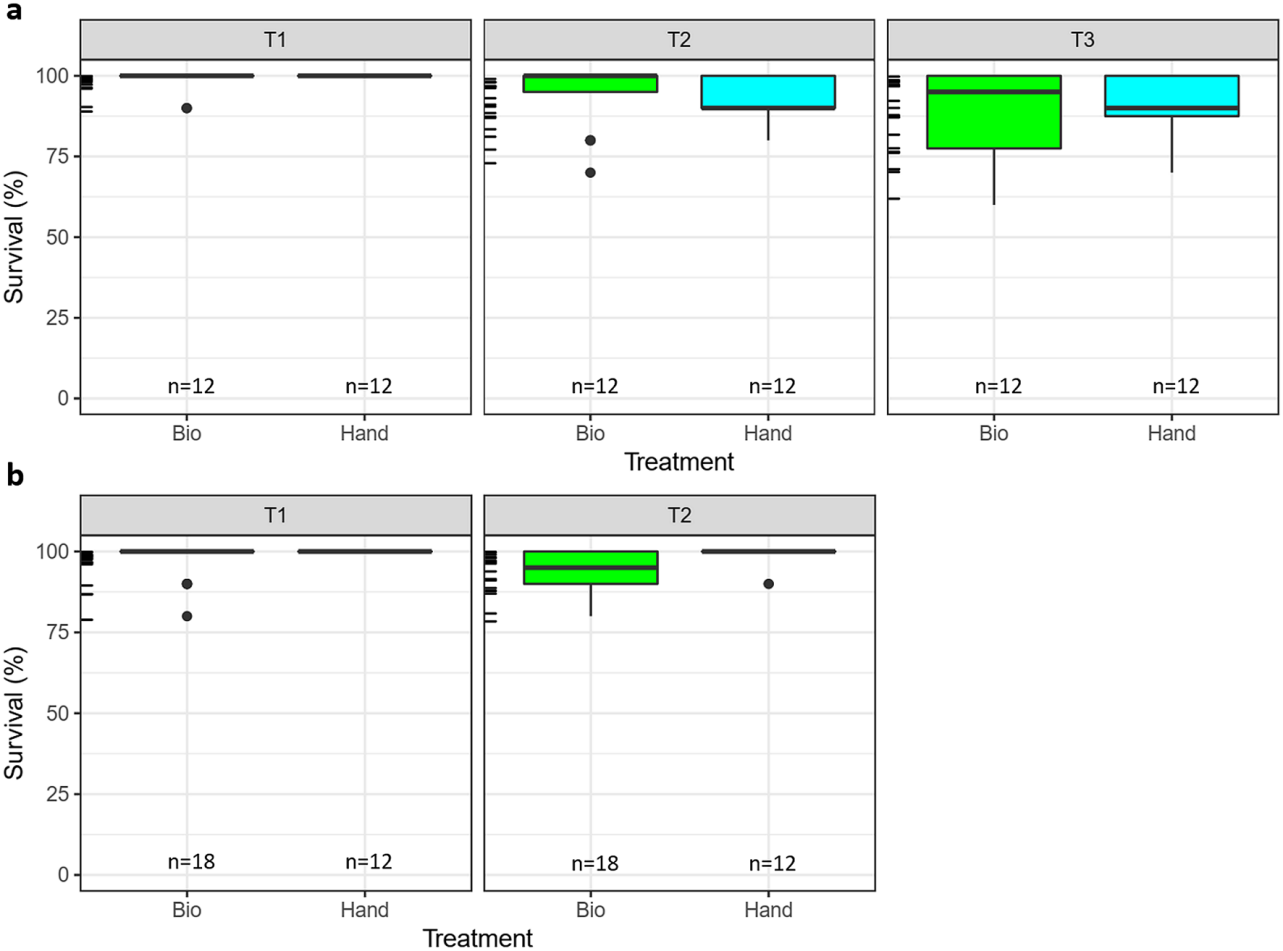
Assay survival success. Survival of *Montipora capitata* mechanically sorted by COPAS Biosorter (“Bio”) and manually hand-sorted (“Hand”) from two replicated viability assays (**a**, **b**). Assay 1 (**a**) measured survival at 0, 18, and 36 h (T1, T2, and T3, respectively). Assay 2 (**b**) measured survival at 0 and 24 h (T1 and T2, respectively). Sample size (n) refers to the number of replicate wells tested in each assay for each treatment; each well contained 10 larvae. Tick marks on the y-axes represent jittered rug plots of the data.

Survival of mechanically-sorted larvae averaged over 90% after 24–36 h and was comparable with rates achieved by careful hand sorting [91 ± 2% vs. 95 ± 2% (mean ± SE), respectively; GLM assay 1: *z* = 0.012, *p* = 0.99; assay 2: *z* = 0.08, *p* = 0.99] (Fig. 1). No significant differences in survival between hand-sorted and mechanically-sorted samples were detected in either assay, although in assay 2, survival after 36 h was significantly lower in both mechanically-sorted and hand-sorted treatments [88% ± 4 and 91% ± 3 (mean ± se), respectively; GLM, *z* = − 2.8, *p* = 0.005].

A small percentage (0.7%) of larvae appeared to disintegrate immediately upon deposition; it was unclear whether those larvae were damaged while passing through the flow cell (i.e. during the ‘flight’) or if they had died prior to the flight but had remained intact in the container prior to being sampled. When only larvae of normal appearance were supplied to the Biosorter, however, survival rates were 100%, with 98% of the larvae remaining morphologically normal post-deposition. Eighty nine percent of the hand-selected, mechanically-sorted larvae demonstrated rotary swimming and searching behaviour almost immediately after the ‘flight’; of those larvae that were not behaving normally after deposition, most resumed swimming with a few minutes, appearing to have just been temporarily ‘stunned’.

The integrity of every preserved embryo tested in the viability assay was maintained. 46 of the 48 embryos were successfully deposited into the wells of a 48-well plate and appeared normal and intact. 2 of the 48 embryos were mis-deposited outside of the wells, but also appeared normal and were returned to the well via pipette.

### Spectral sorting and developmental stage recognition

**Figure 2:**
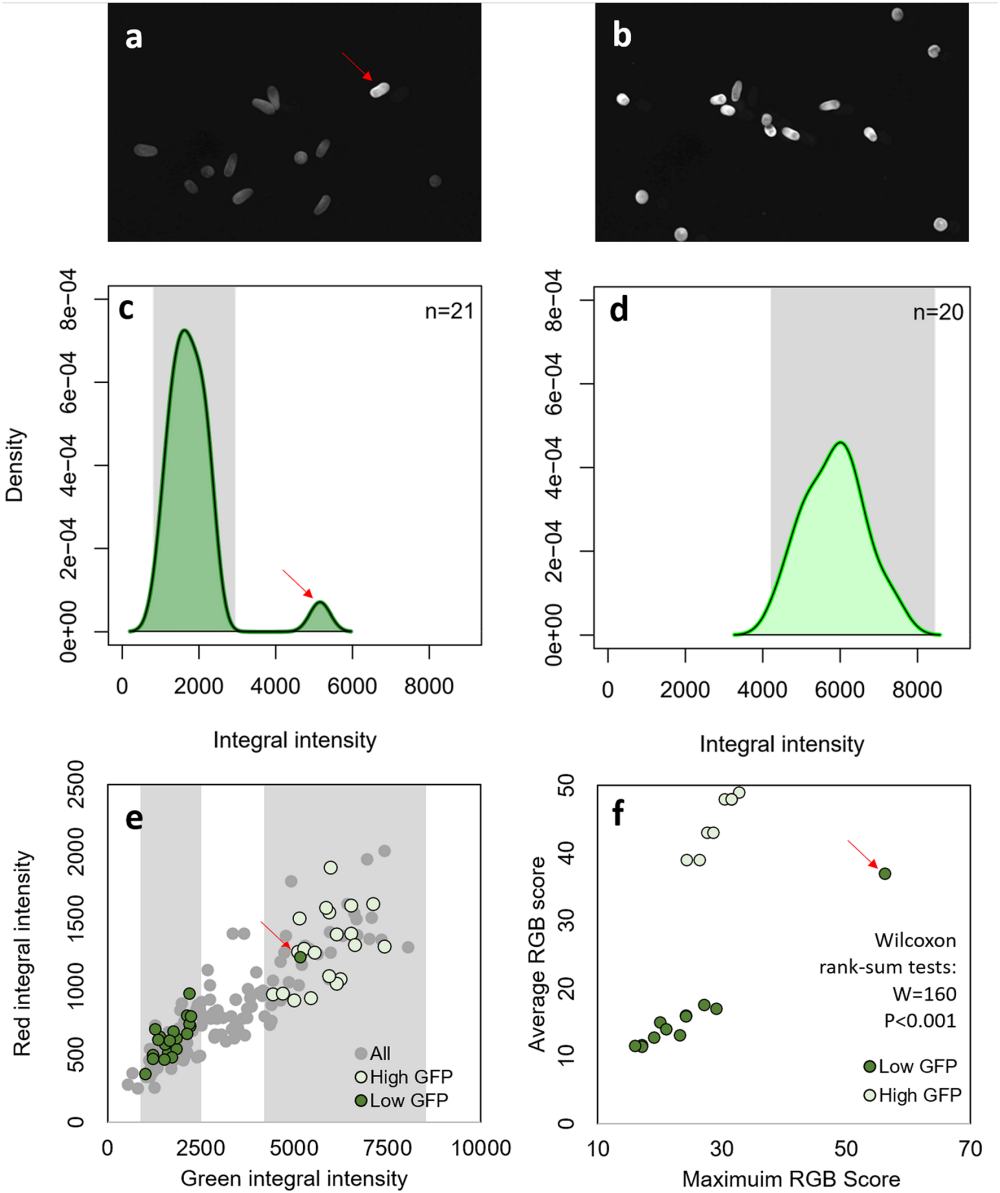
Fluorescence micrographs of live *Montipora capitata* larvae that were sorted by the COPAS Biosorter into low green fluorescent protein (GFP) (**a**) and high GFP (**b**) samples, with corresponding sample density plots (**c**, **d**, respectively). (**e**) All larvae detected in the fluorescence sorting run (gray points) with those sorted into low and high GFP samples identified. (**f**) The average and maximum RGB scores validated through fluorescence microscopy for the sorted larvae. Results of un-paired Wilcoxon rank-sum tests comparing the average and maximum values between the two populations are reported. Grey shaded areas in **c–e** represent the green fluorescence detector integral intensity gates used to sort the larvae into ‘low’ and ‘high’ fluorescence groups. We note that one high-GFP larva was correctly measured but mis-deposited into the low-GFP sample due to the close proximity of the larvae in the flow cell (red arrows in **a**, **c**, **e** and **f**).

**Figure 3:**
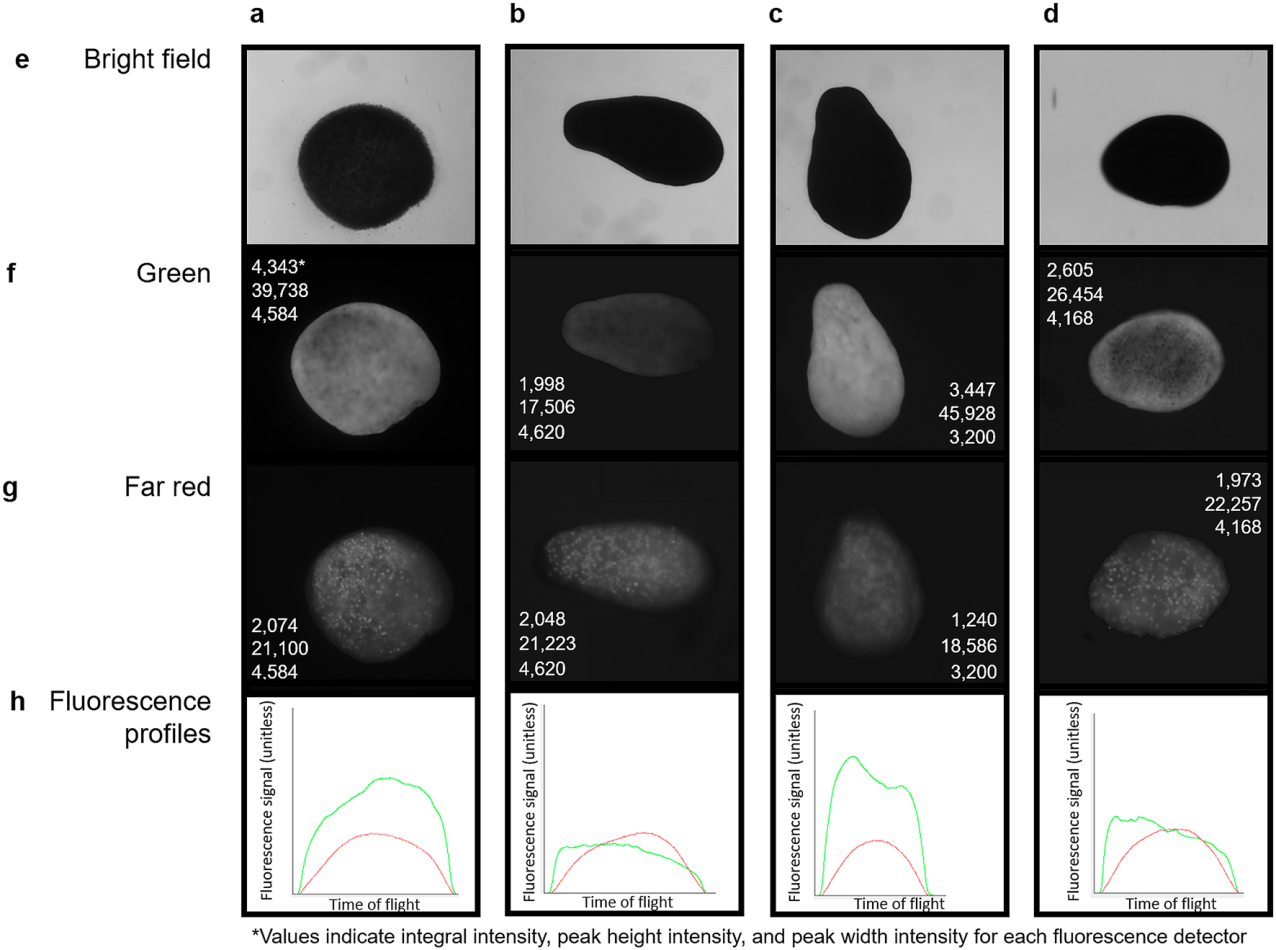
Brightfield (**e**), green (excitation 488 nm) (**f**) and far red (excitation 561 nm) (**g**) fluorescence micrographs, and corresponding fluorescence profiles (**h**) of four live *Montipora capitata* larvae (**a–d**) sorted with the COPAS VISION flow cytometer. Brightfield images (**e**) and fluorescence profiles (**h**) were obtained with the flow cytometer, and the fluorescence micrographs (**f**, **g**) were imaged using fluorescence microscopy. The three values on each fluorescence micrograph indicate the integral intensity, peak height intensity, and peak width intensity for each fluorescence detector. Time of flight in (**h**) has arbitrary units here, but can be used to calculate particle size due to the stable laminar flow and constant velocity of particle movement through the flow cell.

The initial run to capture the population-level variability in green larval fluorescence identified an integral intensity that ranged from 0 to 8,020, with a fairly even spread (Fig. 2e). A low GFP gate (830–2,300) and a high GFP gate (4,300–8,150) around the integral intensity values were established and used as sorting criteria (Fig. 2c–e). Because any particle that did not meet sorting requirements was sent to ‘waste’, 173 particles were run through the flow cytometer before 20 low GFP larvae (Figs. 2a,c, 3b) followed by 20 high GFP larvae (Figs. 2b,d, 3a) were collected. The throughput rate for the spectral sorting was 4.0 s particle^−1^ and it took a total collection time of ~ 12 min.

All larvae but one were correctly sorted based on their fluorescence profile; one highly fluorescent larva was mis-deposited with the low-fluorescent larvae (Fig. 2a,c,e,f) because the particles were too close together in the flow cell to be effectively separated in the deposition droplet. However, the fluorescent profiles of that larva were correctly quantified in the sample output. Validation through fluorescence microscopy indicated that the spectral sorting was effective, and the post hoc analysis of the fluorescence photomicrographs detected statistically significant differences in the average and maximum RGB values between the two groups (Wilcoxon rank-sum tests: W = 160, *p* < 0.001).

**Figure 4:**
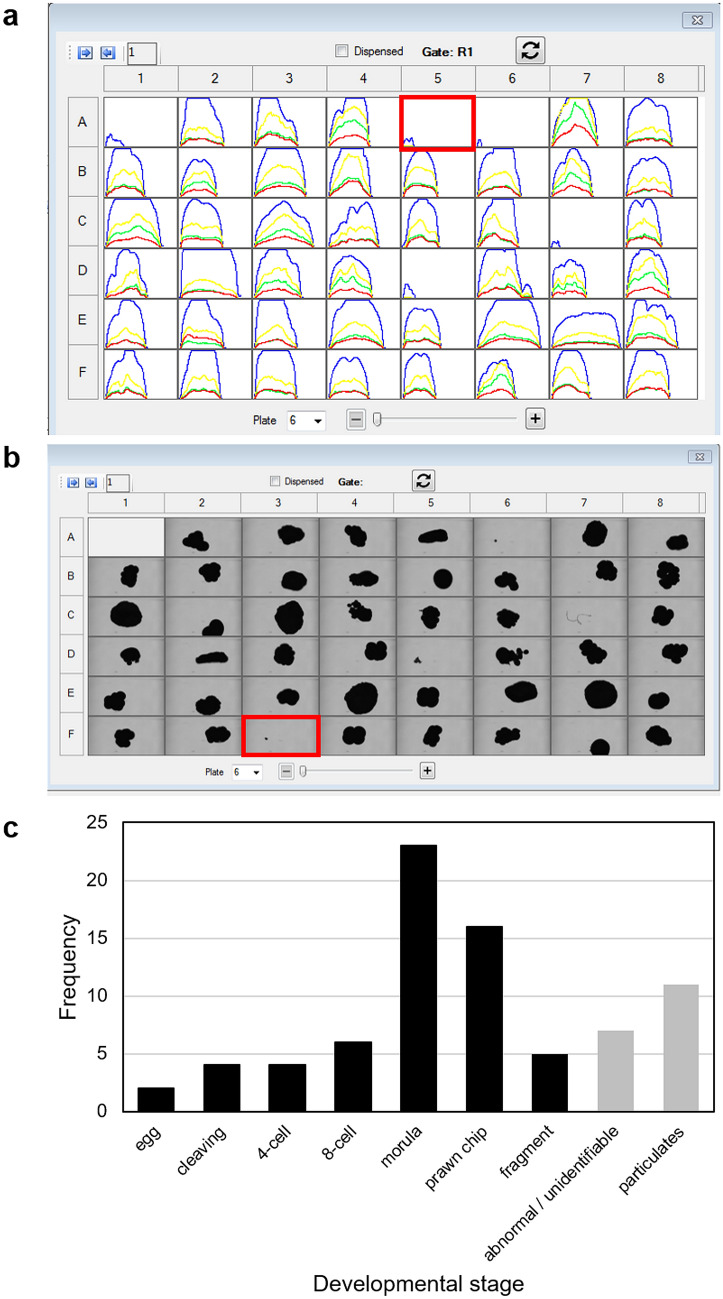
Fluorescence detection profiles (**a**), corresponding brightfield photomicrographs (**b**), and a frequency distribution of the developmental stages (**c**) identified from particles in a sample tested on the COPAS Biosorter. In panel (**a**) blue curves are extinction profiles, and yellow, green and red curves are yellow, green and red fluorescence profiles, respectively. The red box in (**a**) highlights a particle that missed detection but was photographed. The red box in (**b**) identifies a particle that missed the photomicrograph but was detected. Black bars in (**c**) represent clearly identified developmental stages; grey bars represent abnormally shaped or unidentifiable embryos or particulates [as in cells C7 and D5 in (**b**)] in the sample.

Larval fluorescent profiles indicated that GFP was not uniformly distributed throughout an individual larva; the oral end was often more fluorescent than the aboral end (see Fig. 3bf & bh, df & dh), although we note that the orientation of the larva through the flow cell was not possible to control and does not always align with the brightfield image. Red fluorescence (RF), on the other hand, followed a predictable curve suggesting a more uniform distribution of *Symbiodinium* throughout the larvae. The intensity of GFP was also more variable than RF (compare larvae b and c in Fig. 3; Fig. 4a). We also note that a significant positive correlation between the integral intensity of the red and green fluorescence was detected across all larval samples (Pearson’s correlation: *t* = 21.9, df = 149, *p* < 0.0001, R^2^ = 0.87; Fig. S2), as was a correlation between the peak height (maximum intensity) of the red and green fluorescence spectra (Pearson’s correlation: *t* = 16.0, df = 131, *p* < 0.0001, R^2^ = 0.81), and the integral intensities of the green and far red fluorescence (Pearson's correlation: *t* = 8.7, df = 55, *p* < 0.0001, R^2^ = 0.76).

**Figure 5:**
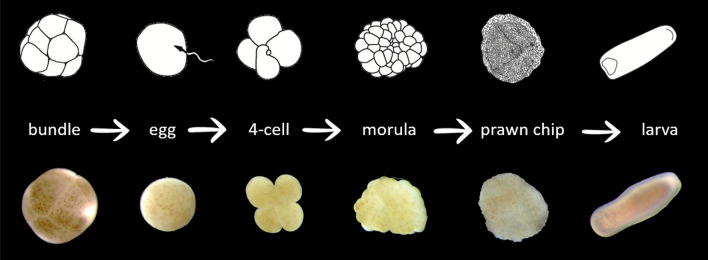
Line-drawn stages (top panel) with corresponding photomicrographs (bottom panel) of *Montipora* spp., from the bundle release during spawning, to the fully developed planula larva. Larvae are approximately 500 µm in length. Photomicrographs of the bundle through prawn chip are *Montipora capitata*; the larva is *Montipora digitata*. Images: C. Randall.

Ninety percent of the embryo samples that were imaged with the COPAS VISION (Fig. 4b) were manually identified through their photomicrographs (Fig. 4c). The particles were assigned to one of 9 categories defined by developmental stage (Figs. 4c, 5) and a frequency distribution of developmental stages present in the mixed sample was generated (Fig. 4c).

## Discussion

Most tropical reef-building corals spawn gametes annually, typically with intraspecific spawning synchrony on one or a few consecutive nights, providing brief access to study early life-history stages^6,7^. When investigating fertilisation, embryogenesis, and larval development, laborious manual counting and sorting has been the norm. Consequently, the early life stages of only a small fraction of the species known to broadcast spawn have been well studied (i.e.^9^). Expanding the scale and diversity of experiments requires techniques that can take advantage of the high fecundity of most broadcast-spawning species, while managing both the buoyant and delicate early stages^21^ and the robust but motile larvae, each of which present their own set of challenges. Here we tested the ability of two large-particle flow cytometers to characterise and sort live and preserved eggs, embryos, and larvae of the Hawaiian coral *Montipora capitata* using spectral data. Both instruments rapidly and accurately counted, measured, and non-destructively sorted viable coral samples based on size and real-time fluorescence detection, offering a promising new tool for investigations with coral propagules, both for basic and applied research^24^.

Understanding the role of intrinsic fluorescence of coral gametes and larvae in coral biology and physiology is an active area of research^23,25–27^, and flow cytometry is well-placed to exploit this fluorescence and other spectral properties for rapid sorting and analysis of fluorescent properties. Fluorescent proteins in corals have many hypothesized physiological and ecological roles, including protecting symbionts under high-light conditions and enhancing light availability under low-light conditions^22,25,28^, protecting the host from oxidative stress^29^, reducing herbivory^30^, and attracting photosymbionts to the larvae^31,32^. The concentration of GFP varies naturally among species and individuals, and throughout embryogenesis and larval development^23,27^. Here we rapidly quantified the patterns of GFP among (Fig. 2) and within (Fig. 3) individual *M. capitata* embryos and larvae. Our testing confirmed that there is a broad distribution of fluorescence intensities among individuals within a spawning population of *M. capitata* from Hawaiʻi (Fig. 2) and suggests that fluorescence is often concentrated around the oral end of the larva (see BF and DF in Fig. 3). Furthermore, a strong positive correlation between the fluorescence intensity of GFP and RF across samples and developmental stages was identified (Fig. 2e, S2), suggesting that *M. capitata* eggs and larvae with higher densities of GPFs also have higher densities of endosymbionts. Because *M. capitata* vertically transmit their endosymbionts, it is unclear whether GFP would act to attract additional symbionts to the larvae of this species^31^; this hypothesis has yet to be experimentally tested in *M. capitata* and warrants further investigation. Regardless, the current study highlights the suitability of large-particle flow cytometry to rapidly advance investigations into the roles that GFPs serve in the embryos and larvae of this and other species.

In response to anthropogenic reef decline, restoration programs are gaining traction globally, although with significant scalability challenges^5,33^. Larger-scale restoration methods using coral spawn harvested from natural slicks and then released onto degraded reefs once they are competent to settle is straightforward^34–36^; but processing, quantifying and describing the composition, developmental stages, and quality of the slick represents a significant challenge and bottleneck in the process. Visual analysis of brightfield images was able to accurately identify different life stages from mixed samples, demonstrating the potential for large-cell imaging flow cytometry to help characterise the components of a spawning slick; however, there was no way to automatically sort individual particles based on data collected from the brightfield photomicrographs in the instrument tested here. Advances in automated image analysis^37^ and millisecond to nanosecond processing times would be required to (i) take the image, (ii) classify the particle in the image using an automated image classification pipeline^14^, and then (iii) communicate the sorting decision to the cytometer using a hybrid software-hardware data management infrastructure in the milliseconds it takes the particle to pass through the flow cell^38^. Regardless, our exploratory investigation demonstrates that particles can be used to quantify sample composition, and combined with technological advancements, such as the intelligent image-activated cell sorting machine-intelligence technology proposed by Isozaki et al.^38^, has the potential to be applied to real-time image-based sorting of large particles, including coral spawn.

Another technique being applied in coral restoration is the cryopreservation of gametes, embryos and larvae^24,39–41^. Currently, 1,000’s of coral larvae can be frozen in an hour with high-throughput cryopreservation methods; this process encases the embryos in a vitrified bead of cryoprotectant solution that can be placed in long-term storage or thawed, assessed, and settled^39^. The thawing methods used, to date, have involved single lasers that can only process a few beads per minute. However, high throughput laser-warming technology married with large-particle flow cytometry of thawed and live-dead stained larvae could make this suite of techniques reef-restoration ready.

Large-particle flow cytometry has also been applied to the screening and sorting of microencapsulated particles, cells, and organisms, such as fungal spores encapsulated in calcium alginate beads^18^. A recent investigation that immobilised several species of coral propagules in an agarose gel found that survival of immobilised larvae was high, and that the encapsulation process did not significantly impede larval settlement after immobilisation^42^. A work flow could be developed whereby the output from automated encapsulation feeds directly into a large-particle flow cytometer to measure and sort individually-encapsulated particles. Alternatively, the flow cytometer could be used to deposit larvae directly into an encapsulation medium for down-stream applications.

With an egg diameter of approximately 500 µm^43^ and a larval length of ~ 750 µm, *Montipora capitata* propagules were well suited to the 1,000 µm fluidics and optics core assembly (FOCA) unit tested here. Coral propagules span a considerable size range, however, from ~ 80 µm for the small broadcast spawning *Lobactis scutaria* (syn. *Fungia scutaria*)^44^ to the large ~ 1,500–2000 µm planula larvae released by some brooders, such as *Pocillopora* spp.^45^ and *Favia* spp.^46^. The instruments tested here can comfortably accommodate particles from 20 to 1,500 µm in diameter, across their range of flow-cell sizes (up to 2000 µm), and are thus compatible with the vast majority of broadcast spawning coral species, and many brooding species. Other marine organisms with adult or larval stages within this size range, such as zooplankton tested by Henzler et al.^17^, would also be suitable for study with these instruments.

In this period of rapid ocean warming, large-scale and high-throughput methodologies are more important than ever, to advance basic and applied research into the early life-history stages of corals quickly and efficiently. We tested one such method, and conclude that gentle, large-particle flow cytometry can rapidly and efficiently sort live coral propagules based on spectral properties and size. Further development of this technique offers rapid characterisation and quantification of both wild-caught and laboratory-cultured coral propagules.

## Methods

### Coral spawning and larval rearing

*Montipora capitata* Dana (1846) is a broadcast-spawning scleractinian, found across the tropical Pacific and is very common in Hawaiʻi^47^. This species typically releases gamete bundles containing sperm and 8–10 (500 µm diameter) eggs with parentally-derived (vertically transmitted) dinoflagellate symbionts (Symbiodiniaceae)^43^. Fecund colonies of *Montipora capitata* were collected in June, July and August, a few days prior to the predicted spawn dates for each of the spawning events. Colonies were collected from throughout Kāneʻohe Bay to ensure a wide diversity of genotypes contributed to the coral cultures. Colonies were transported to the Hawaiʻi Institute of Marine Biology in Kāneʻohe Bay where they were maintained in outdoor aquaria with flow-through filtered seawater under ambient light and temperature (midday ~ 26 to 28 °C) conditions. Aquaria were fitted with shade cloths to match irradiance levels in the shallow tanks with intensities measured on the reef. On the evenings of 12–13 July 2018 and 11–12 August 2018 (1–2 nights after the new moon), approximately 2 h before the predicted spawning, the colonies (n = 15–20) were placed into 20 L floating plastic flowerpots with running seawater. At approximately 21:00 (90 min after sunset), the colonies began spawning gamete bundles. Bundles were gently collected from the surface of the aquaria via 1.5 mL plastic transfer pipettes and transferred to scintillation vials with 4.9 mL of 0.2 µm filtered seawater. One of the most effective ways to fertilise this species is through bundle-bundle crosses^40^. Typically, one bundle each, from two parent colonies, was placed in each vial with approximately 100 µL of seawater, to bring the total volume to 5.0 mL. Each bundle contained an average of 9.6 eggs (± 1.4 SD, n = 9), for an average of 19 eggs per vial. Bundles were left to break apart naturally and allow fertilization to occur. Sperm concentrations within the vials were approximately 1.2 × 10^7^ as measured from haphazard samples collected from a subset of vials and analysed on a Hamilton Thorne CEROS II automatic sperm analysis system. Approximately 4 h after fertilisation, 10 mL of 0.2 µm filtered seawater were added to each vial to dilute the sperm and bring the total volume to 15 mL. Vials were placed in an incubator set at 26 °C overnight and allowed to develop. Embryos were also reared in 50 mL Falcon tubes at a similar density and sperm concentration, and concurrent mass cultures were set-up in flow-through larval rearing cones to rear large numbers of larvae; six brine shrimp rearing cones (Pentair, Cary, NC USA) were stocked at a target density of ~ 1 larva ml^−1^.

Cleaving was observed beginning ~ 03:30 h after fertilisation; 4-cell embryos and prawn chips were observed ~ 05:30 and ~ 14:30 h after fertilisation, respectively. Throughout the night and next day, samples of unfertilised eggs, embryos undergoing initial cleavage, 4-cell embryos, 8-cell embryos, morulae, prawn chips, and early larvae were collected from the scintillation vials or Falcon tubes and fixed in 4% paraformaldehyde in 0.2 µm filtered seawater and placed at 0 °C. Live larvae were maintained in larval rearing cones until transport.

### Larval transport

On 14 Aug 2018, live *M. capitata* larvae (2–3 days old) were packed in 500 mL polyethylene containers with 0.2 µm filtered seawater at a density of approximately 0.4 larvae per ml^48^. Care was taken to minimize trapped air in the sealed containers to reduce shear stress during transport from the water–air interface^49^. Six containers (total volume of 3 L with ~ 1,200 larvae) were packed into each of two small coolers lined with an insulating thermal blanket. To monitor air temperature inside the coolers during transit, a standard aquarium thermometer was placed in each cooler with a lead to a digital display outside the cooler. Three HOBO onset data loggers were submerged in haphazardly selected containers in each cooler to monitor seawater temperature every 5 min during transport (Figure S1). Within each cooler, there was a 750 mL rubber hot water bottle that was filled with boiling water and used to maintain the internal temperature of each cooler, when the temperature dropped below approximately 25 °C. Coolers with larvae were transported in the pressurized passenger cabin on board commercial flights from Oahu to Los Angeles to Boston (~ 12 h in transit). Upon arrival at Union Biometrica (Holliston MA, USA) larval containers were transferred to, and maintained in, a temperature-controlled incubator at 27 °C. All corals were collected and transported under the State of Hawaiʻi Department of Land and Natural Resources Division of Aquatic Resources Special Activity Permit no. SAP2019-16, Amendment 1, granted to the Hawaiʻi Institute of Marine Biology.

### Flow-cytometry trials

Particle sorting of live and preserved coral embryos and larvae was tested using two commercially available large-particle (up to 2000 µm) flow cytometers that employ flow spectroscopy: COPAS (Complex Object Parametric Analyser and Sorter) Biosorter and COPAS VISION (Union Biometrica, Inc.)^20^.

### COPAS Biosorter

The COPAS Biosorter was tested with a 1,000 µm fluidics and optics core assembly unit (FOCA; 1 mm^2^ flow cell). The 1,000 µm FOCA was selected because mature *M. capitata* eggs/embryos measure approximately 500 µm in diameter^43^, with elongated larvae reaching up to ~ 750 µm in length. Sample delivery was tested with a 1 L sample cup and a 50 mL Falcon tube. Laminar flow of particles through the flow cell was achieved with the delivery of sheath fluid supplied with artificial seawater made using deionized water and Instant Ocean Reef Crystals. A downstream pneumatic diverter (sorting) valve gently dispensed the samples in a fluid droplet (~ 10 µL); sorted (‘kept’) particles were dispensed into a collection container (well plate, petri dish or 50 mL Falcon tube) and waste ( ‘discarded’) particles, which were recoverable and viable, were gently deposited into a waste collection container with filtered seawater.

The optics assembly unit was tested with two excitation solid state lasers (488 and 561 nm), a photodiode detector for measuring extinction and time of flight (to estimate particle size), and photomultiplier tubes for fluorescence emission detection in the green (bandpass 525/50), yellow (bandpass 543/22), red (bandpass 586/20) and far red (bandpass 680/42) regions of the visible light spectrum. A 488 nm excitation laser with a power of 100 mW was chosen to excite the corals’ cyan-green fluorescent proteins (GFP), which emit fluorescence in the green (~ 500 nm, green detector; see *Spectral Sorting* below;^25,27,50^. The 561 nm excitation laser with a power of 50 mW was selected to measure the endosymbiotic Symbiodiniaceae (sensu^51^), which fluoresce in the red (see *Spectral Sorting* below)^50^. The measurements for the analysed objects include the integrated values for extinction and fluorescence, as well as positional information for the distribution of the signals across the object (Figs. 3h, 4a) and are referred to as the profile for the object.

### COPAS VISION

The COPAS VISION was outfitted with the same flow cell and optics assembly unit as the COPAS Biosorter, but also has an in-line brightfield camera with a fixed focal length that imaged each particle as it passed through the flow cell, prior to sorting. The 5 megapixel CMOS camera used a 740 nm illumination, 2 × objective, capable of 350 frames per second with a 1 µs exposure time. Sample delivery was tested with a gentle 50 mL rotating sample cartridge and a 50 mL Falcon tube.

### Throughput rates

The performance of live and preserved eggs, embryos and larvae, across developmental stages (Fig. 5) was evaluated through a series of runs on two flow cytometers (Table S1). The samples included live 4–6 day old planula larvae, and preserved unfertilised eggs, early-stage embryos (4-cell to morulae), prawn chips, and larvae. Samples of different densities, and using different delivery methods and receptacles (15 ml tube, 50 ml tube, 15 ml rotating sample cartridge) were tested, and the run-time and particle counts were recorded for each test (Table S1). Throughput rates, defined as the number of particles processed per second, were calculated for each run.

### Larval viability assays

To evaluate the effect of a COPAS Biosorter ‘flight’ on the viability of live larvae, survival rates of mechanically-sorted and hand-sorted larvae were compared in two replicate assays. Ten larvae per well were sorted into polystyrene six-well culture plates (n = 12–18 replicate wells per treatment), either using the COPAS Biosorter or pipetted by hand. All larvae came from the same source container and were sorted into wells with 10 mL of 0.2 µm filtered seawater. The larvae were assessed immediately after sorting and again after 18–36 h, and the numbers of live and dead larvae were recorded and compared between the two techniques. Larval survival by time was modelled using a generalized linear model fit with a binomial distribution and a logit link function. Model assumptions and diagnostics were checked with residual plots. All models were run using package ‘lme4’^52^ in R^53^.

While we took care to minimise selection bias in the assays, it is possible that normal swimming larvae were preferentially hand-sorted, whereas the Biosorter was completely unbiased, selecting particles only by size and broad fluorescence gating; thus, it is possible that some mechanically-sorted particles were larval fragments or were in poor condition prior to dispensing. To account for this bias, an additional mechanical trial was conducted with 50 hand-selected, swimming, normal looking larvae that were carefully selected and placed into a 50 mL Falcon tube with 0.2 um filtered seawater (‘Individual larval viability assay’ in Table S1). The larval sample was introduced to the COPAS Biosorter and mechanically deposited into a 48-well plate with one larva per well. After dispensing, each larva was assessed under a standard dissection microscope and its condition was recorded. Because this trial was observational, no statistical analyses were undertaken.

In addition to the live samples, the integrity of preserved embryos that passed through the flow-cytometer was evaluated. Preserved morulae and prawn-chip samples were used due to the fragility and vulnerability of blastomeres that compose the naked embryo. One particle per well was deposited into a 48-well plate from a mixed sample, using the COPAS VISION. The integrity of each embryo was evaluated after sorting using a standard dissection microscope.

### Spectral sorting and developmental stage recognition

*Montipora capitata* transmit their endosymbiotic dinoflagellates through spawned eggs [i.e. are vertical transmitters^43^; therefore, their eggs, embryos and larvae contain photosynthetic Symbiodiniaceae cells, which, when excited by green wavelengths ~ 560 nm, emit fluorescence in the far red spectrum (~ 685 nm)]. Adult and larval *Montipora capitata* also contain pigments that are (at least partially) homologous to cyan-green fluorescent proteins (GFP), common in anthozoans^22,25,27^.

To evaluate the ability of the flow cytometer to sort larvae based on real-time fluorescence detection, a sample of larvae was first run through the flow cytometer to optimise detection and identify the range of fluorescence intensities present within the sample population. Then, gates were established, based on the integral of the fluorescence intensity from the green detector, to capture “low” and “high” levels of GFP, and subsequently were used as sorting criteria. A sample of larvae was run through the COPAS Biosorter, to sort 20 low-GFP larvae and 20 high-GFP larvae (Table S1). A ZEISS Axiovert 200 fluorescence microscope with an arc lamp light source, GFP filterset (bandpass 525/50), and a camera (ZEISS Axiocam 503) was used to image the sorted larvae, and the accuracy of the fluorescence-based sorting by the flow cytometer was validated using ImageJ (https://imagej.nih.gov/ij/). Fluorescence photomicrographs were evaluated using the ‘RGB Measure’ plugin, by outlining each larva and measuring an average and a maximum total RGB score. Unpaired two-sample Wilcoxon rank sum tests were used to compare the average and maximum RGB scores between the ‘low’ and ‘high’ fluorescent samples, to validate the fluorescence sorting; non-parametric tests were used because the data did not meet assumptions of parametric tests. Data analyses were performed using the ‘wilcox.test’ function in base R^53^.

To explore whether the early developmental stages of coral embryos could be identified from the COPAS VISION brightfield photomicrographs, preserved eggs and embryos of various stages were mixed in a single sample. Approximately 100 particles were run through the flow cytometer and deposited into wells of 48-well plates. The brightfield photomicrographs were then used to attempt manual classification of each particle by developmental stage.

## Supplementary information

Supplementary information.
