# Supplementary material for: Rapid counting and spectral sorting of live coral larvae using large-particle flow cytometry
Source: Sci Rep. 2020 Jul 31;10:12919. doi: 10.1038/s41598-020-69491-0 (PMC7395729; doi:10.1038/s41598-020-69491-0)
Supplement: Supplementary file 1 — Supplementary information. [file 41598_2020_69491_MOESM1_ESM.docx]

# Supplementary material: Rapid counting and spectral sorting of live coral larvae using large-particle flow cytometry

Carly J. Randall^1*^, Justin E. Speaks^2^, Claire Lager^3,4^, Mary Hagedorn^3,4^, Lyndon Llewellyn^1^, Rock Pulak^5^, Julia Thompson^5^, Line K. Bay^1^, David Mead^1^,

Andrew J. Heyward^6^, Andrew P. Negri^1^

^1^Australian Institute of Marine Science, Townsville QLD, Australia

^2^Townsville QLD, Australia

^3^ Hawaiʻi Institute of Marine Biology, Kāneʻohe HI, USA

^4^Center for Species Survival, Smithsonian Conservation Biology Institute, Front Royal VA, USA

^5^ Union Biometrica, Inc. Holliston MA, USA

^6^Australian Institute of Marine Science, Indian Ocean Marine Research Centre, University of Western Australia, Crawley WA, Australia

*Correspondence to: c.randall@aims.gov.au

# Supplementary Tables

Supplementary Table 1. Summary of all flow cytometer sample runs undertaken with *Montipora capitata* preserved and live developmental samples.

| **Flow Cytometer** | **Run** | **Description** | **State** | **Sample type** | **Sample delivery system** | **Collection container** | **Approximate starting density** | **Time (sec)** | **# particles** | **# particles second^-1^** |
| --- | --- | --- | --- | --- | --- | --- | --- | --- | --- | --- |
| COPAS Biosorter | Initial tests to define larval parameters | Ran larvae through to identify distribution of particle size and fluorescence for gating of future runs. Didn't sort; went to waste container. Sheath was DI water so run was destructive. | Live | Larvae | 50 ml tube | None | 1-5/ml | 370 | 86 | 0.2 |
|  | Sorting larvae by low fluorescence | Run to collect 20 larvae from R12 gate (low fluorescence). | Live | Larvae | 50 ml tube | 6-well plate | 1-5/ml | 540 | 107 | 0.2 |
|  | Sorting larvae by high fluorescence | Run to collect 20 larvae from R11 gate (high fluorescence). Sorting here was faster because a higher proportion of larvae were within the high fluorescence gate. | Live | Larvae | 50 ml tube | 6-well plate | 1-5/ml | 137 | 66 | 0.5 |
|  | First larval viability assay, plate 1 | Sorted 10 larvae per well using size only as criteria (gating by time of flight and extinction). | Live | Larvae | 50 ml tube | 6-well plate | 1-5/ml | 298 | 90 | 0.3 |
|  | First larval viability assay, plate 2 |  | Live | Larvae | 50 ml tube | 6-well plate | 1-5/ml | 386 | 80 | 0.2 |
|  | Second larval viability assay, plate 1 | Sorted 10 larvae per well using size only as criteria (gating by time of flight and extinction). | Live | Larvae | 15 ml tube | 6-well plate | 5-10/ml | 207 | 85 | 0.4 |
|  | Second larval viability assay, plate 2 |  | Live | Larvae | 15 ml tube | 6-well plate | 5-10/ml | 408 | 104 | 0.3 |
|  | Second larval viability assay, plate 3 |  | Live | Larvae | 15 ml tube | 6-well plate | 5-10/ml | 192 | 81 | 0.4 |
|  | Individual larval viability assay | Sorted 1 larva per well using size only as criteria (gating by time of flight and extinction). All larvae were hand selected as healthy and swimming normally, prior to going into the sample. | Live | Larvae | 50 ml tube | 48-well plate | <1/ml | 489 | 61 | 0.1 |
|  | Bulk test | Sorted 100 larvae into a petri dish using size only as criteria (gating by time of flight and extinction). | Live | Larvae | 50 ml tube | petri dish | 1-5/ml | 271 | 90 | 0.3 |
| COPAS VISION | Initial tests to define larval parameters | Ran larvae through to identify distribution of particle size and fluorescence for gating of future runs. Didn't sort; went to waste container. Sheath was DI water so run was destructive. | Live | Larvae | 50 ml tube | None | 1-5/ml | 809 | 167 | 0.2 |
|  | Initial test to define preserved prawn-chip parameters | Ran sample through to identify distribution of particle size and fluorescence for gating of future runs. Didn't sort; went to waste container. Sheath was DI water so run was destructive. | Preserved | Prawn chips | 15 ml rotating sample cartridge | None | 1-5/ml | 267 | 52 | 0.2 |
|  | Prawn chip viability | Sorted 1 preserved prawn chip per well to evaluate how well they remained in-tact through a flight. | Preserved | Prawn chips | 15 ml rotating sample cartridge | 48-well plate | 1-5/ml | 313 | 28 | 0.1 |
|  | Morulae viability, plate 1 | Sorted 1 preserved morula per well to evaluate how well they remained in-tact through a flight. | Preserved | Morulae | 15 ml rotating sample cartridge | 48-well plate | 1-5/ml | 195 | 55 | 0.3 |
|  | Morulae viability, plate 2 |  | Preserved | Morulae | 15 ml rotating sample cartridge | 48-well plate | 1-5/ml | 301 | 26 | 0.1 |
|  | Larval fluorescence, plate 1 | Sorted 1 larva per well to confirm fluorescence profiles measured in the flow cytometer with fluorescence microscopy | Live | Larvae | 15 ml rotating sample cartridge | 48-well plate | 1-5/ml | 111 | 39 | 0.4 |
|  | Larval fluorescence, plate 2 |  | Live | Larvae | 15 ml rotating sample cartridge | 48-well plate | 1-5/ml | 59 | 12 | 0.2 |
|  | Larval fluorescence, plate 3 |  | Live | Larvae | 15 ml rotating sample cartridge | 48-well plate | 1-5/ml | 408 | 57 | 0.1 |
|  | Initial tests to define unfertilised egg parameters | Ran sample through to identify distribution of particle size and fluorescence for gating of future runs. Didn't sort; went to waste container. Sheath was DI water so run was destructive. | Preserved | Eggs | 15 ml rotating sample cartridge | None | 200/ml | 130 | 386 | 3.0 |
|  | Comparison of GFP in eggs with morulae and larvae | Preserved unfertilized egg run to evaluate population-level GFP peaks in eggs | Preserved | Eggs | 15 ml rotating sample cartridge | None | 50/ml | 189 | 320 | 1.7 |
|  | Mixed preserved sample | Preserved morulae, gastrulae, and larvae to assess brightfield microscopy assessment of developmental stage | Preserved | Mixed | 15 ml rotating sample cartridge | None | 20/ml | 962 | 227 | 0.2 |

# Supplementary Figures


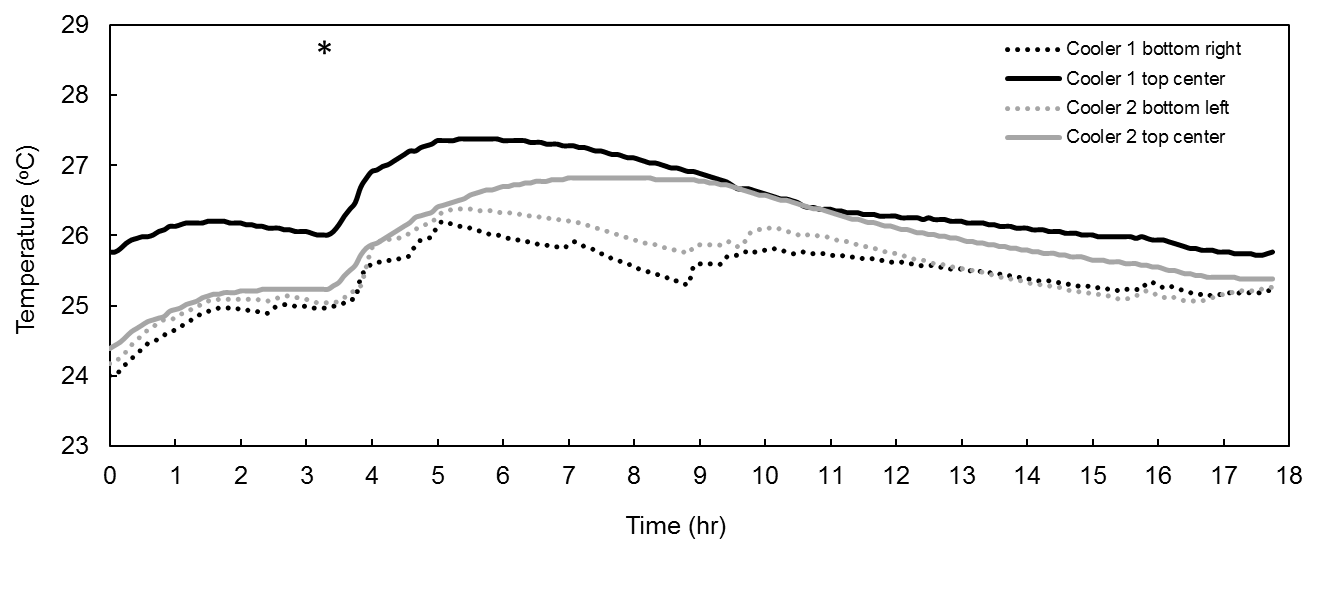


Supplementary Figure 1. Temperature records in the larval containers during transit, as measured by Hobo onset data loggers at five-minute intervals. The asterisk indicates the

introduction of a hot water bottle to each cooler prior to the trans-Pacific commercial flight.


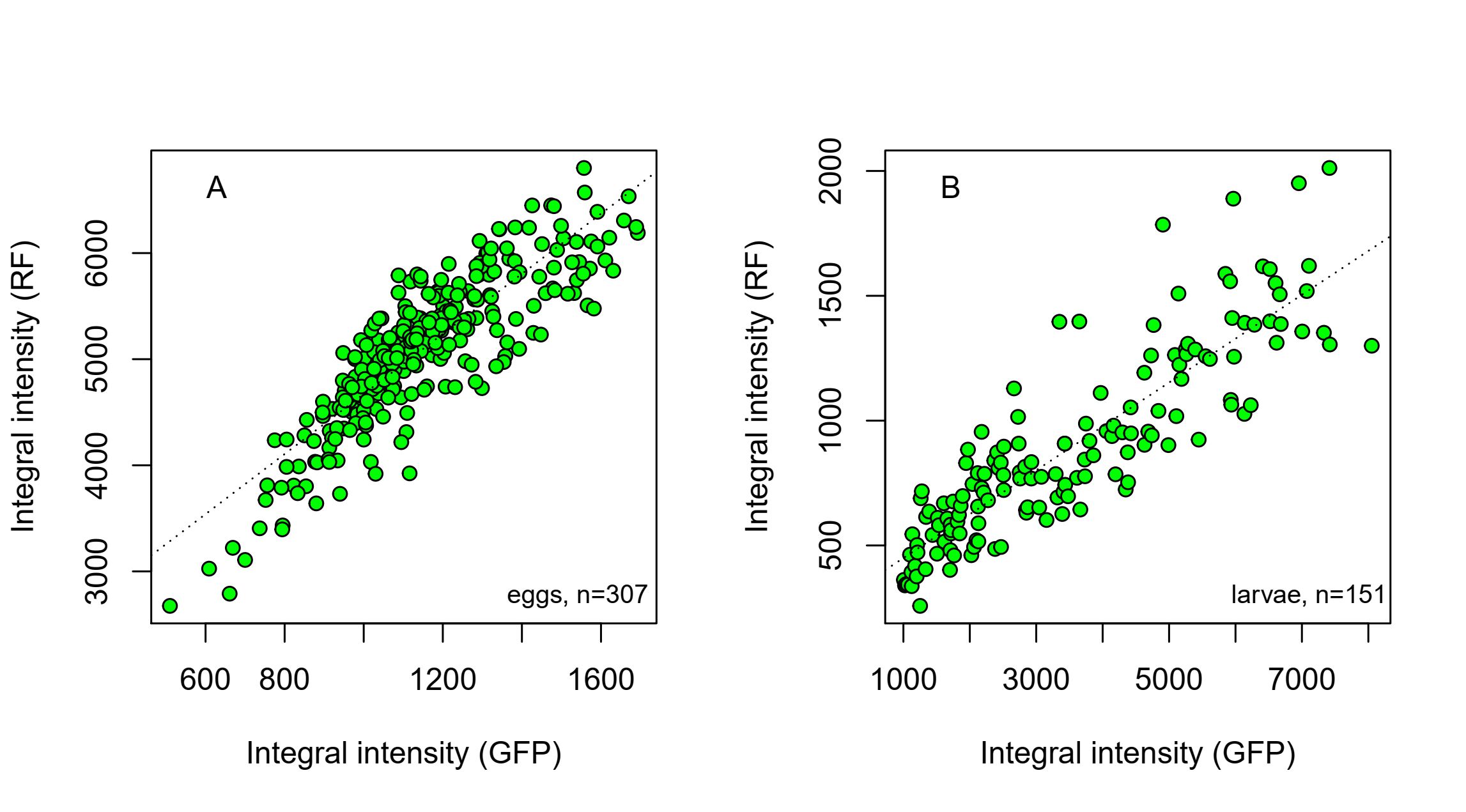


Supplementary Figure 2. Green fluorescence (GFP) integral intensity plotted against red fluorescence (RF) integral intensity for a sample of eggs and larvae measured with the COPAS flow cytometers.
